# Human Vγ9Vδ2 T cell expansion and their cytotoxic responses against cholangiocarcinoma

**DOI:** 10.1038/s41598-024-51794-1

**Published:** 2024-01-14

**Authors:** Piamsiri Sawaisorn, Ahmed Gaballa, Kween Saimuang, Chaniya Leepiyasakulchai, Sakaorat Lertjuthaporn, Suradej Hongeng, Michael Uhlin, Kulachart Jangpatarapongsa

**Affiliations:** 1 Department of Clinical Microscopy, Faculty of Medical Technology, Mahidol University, Nakhon Pathom, Thailand; 2 Center for Research Innovation and Biomedical Informatics, Faculty of Medical Technology, Mahidol University, Nakhon Pathom, Thailand; 3 Division of Hematology and Oncology, Department of Pediatrics, Faculty of Medicine Ramathibodi Hospital, Mahidol University, Bangkok, Thailand; 4 Department of Clinical Science, Intervention and Technology, Karolinska Institutet, Stockholm, Sweden; 5 Department of Clinical Microbiology and Applied Technology, Faculty of Medical Technology, Mahidol University, Nakhon Pathom, Thailand; 6 Department of Applied Physics, Royal Institute of Technology, Stockholm, Sweden; 7 Department of Clinical Immunology and Transfusion Medicine, Karolinska University Hospital, Huddinge, Sweden

**Keywords:** Tumour immunology, Immunotherapy, Lymphocyte activation

## Abstract

Human Vγ9Vδ2 T lymphocytes are regarded as promising effector cells for cancer immunotherapy since they have the ability to eliminate several tumor cells through non-peptide antigen recognition. However, the cytotoxic function and the mechanism of Vγ9Vδ2 T cells leading to specific killing of cholangiocarcinoma cells are yet to be confirmed. In this study, we established a protocol for ex vivo expansion of Vγ9Vδ2 T cells from healthy donors’ peripheral blood mononuclear cells by culture with zoledronate and addition of IL-2, and IL-15 or IL-18 or neither. Testing the cytotoxic capacity of cultured Vγ9Vδ2 T cells against cholangiocarcinoma cell lines showed higher reactivity than against control cells. Surface expression of CD107 was detected on the Vγ9Vδ2 T cells, suggesting that these cells limit in vitro growth of cholangiocarcinoma cells via degranulation of the perforin and granzyme pathway. Analysis of molecular signaling was used to demonstrate expression of pro- and anti-survival genes and a panel of cytokine genes in Vγ9Vδ2 T cells. We found that in the presence of either IL-15 or IL-18, levels of caspase 3 were significantly reduced. Also, IL-15 and IL-18 stimulated cells contained cytotoxicity against cholangiocarcinoma cells, suggesting that stimulated Vγ9Vδ2 T cells may provide a feasible therapy for cholangiocarcinoma.

## Introduction

Cholangiocarcinoma, a malignant cancer which initiates from the epithelial cells lining the bile ducts, is the second most common primary hepatic malignancy^1–3^. It is classified as being either intrahepatic or extrahepatic^4,5^. In Southeast Asia, especially in Thailand, cholangiocarcinoma is common where a high incidence in the northeast region is correlated with infections by the local liver fluke, *Opisthorchis viverrini*^6–9^. According to epidemiologic studies, the worldwide incidence and mortality of this cancer continue to rise^10,11^. At present, surgical resection is the only curative treatment selection for patients and even with this, recurrences are reported^12–15^. Therefore, the identification of novel non-surgical therapies is essential for improving outcomes in patients with cholangiocarcinoma.

Because the immune response can be highly specific, it is hoped that tumor-specific immunity may be used to selectively eradicate tumors without injuring the patient^13,16^. Several immunotherapeutic approaches in cholangiocarcinoma are being actively investigated. These include peptide-based and dendritic cell (DC)-based vaccines, and antibody and adoptive cell immunotherapy. One of these interesting strategies relies on adoptive cell immunotherapy, in which immune effectors cells (such as T cells) are used for treatment of cancer patients with cholangiocarcinoma^17–19^. For instance, cholangiocarcinoma patients who receive a T cell transfer in combination with dendritic cells which were pulsed with a cholangiocarcinoma cell lysate have a lasting response and overall survival of 95.5 months^20^. A similar result has been found in patients with metastatic cholangiocarcinoma who received T cell-based adoptive immunotherapy combined with cetuximab, an epidermal growth factor receptor (EGFR) inhibitor^21^. However, the efficacy of T cell immunotherapies still has limitations because many tumor antigens are only weakly immunogenic. The immune cell may be unable to detect malignant cells and becomes tolerant to further tumor growth and metastasis.

Replacing of conventional T cell therapies might include the use of a subset of lymphocytes, γδ T lymphocytes, an exclusive population of T lymphocyte that express the γδ T cell receptor^22^. Interestingly, many research studies have indicated that tumor cells can be recognized by Vγ9Vδ2 T lymphocytes, the major subset of γδ T cells in peripheral blood, through their expression of specific receptors for mediating anti-tumor responses and controlling tumor development. Vγ9Vδ2 T cells mediate anti-tumor immunity via several evident pathways such as the secretion of proinflammatory cytokines, and cell-to-cell contact-dependent lysis through a natural killer-like pathway or a T cell receptor-dependent pathway^23^. γδ T cells have been shown to devastate cells of many different types of tumor cell lines, including B cell lymphomas, multiple myeloma, and solid tumors of kidneys, colon, prostate and breast. The unique anti-tumor activities of γδ T cells call attention to their potential as candidates for cancer immunotherapy^24,25^. Two possible strategies for Vγ9Vδ2 T cell-based immunotherapy are i) adoptive cell transfer of in vitro expanded γδ T cells, and ii) in vivo therapeutic application of γδ-stimulating phosphoantigens or nitrogen containing bisphosphonates with low-dose recombinant interleukin-2 (rIL-2)^26^. Currently, use of Vγ9Vδ2 T cells as a therapeutic appliance has been studied in both solid tumors and hematologic malignancies. However, studies of Vγ9Vδ2 T cells in cholangiocarcinoma immunotherapy are limited. We found no published literature about the functional assessment of Vγ9Vδ2 T cells against cholangiocarcinoma target cells. Thus, the efficacy of Vγ9Vδ2 T cell-based immunotherapy against cholangiocarcinoma needs to be evaluated.

In the present study, we showed that expanded Vγ9Vδ2 T cells recognized and efficiently killed cholangiocarcinoma cells. Pretreatment of cholangiocarcinoma cell lines with zoledronate can induce anti-tumor activity of Vγ9Vδ2 T cells. Our investigations revealed that the Vγ9Vδ2 T cell cytotoxicity was largely dependent on degranulation via the perforin and granzyme pathway. Importantly, we compared three different stimulation protocols for Vγ9Vδ2 T cells derived from the peripheral blood mononuclear cells (PBMCs) of healthy donors using zoledronate stimulated with cytokines IL-2, IL-15, and IL-18. In vitro characterization of expanded Vγ9Vδ2 T cells was based on data from phenotype, cytokine and apoptotic profiles of the cells. These data provide evidence that Vγ9Vδ2 T cells may be a promising candidate for adoptive immunotherapy against cholangiocarcinoma.

## Results

### Immunophenotyping of Vγ9Vδ2 T cell cultures

**Figure 1:**
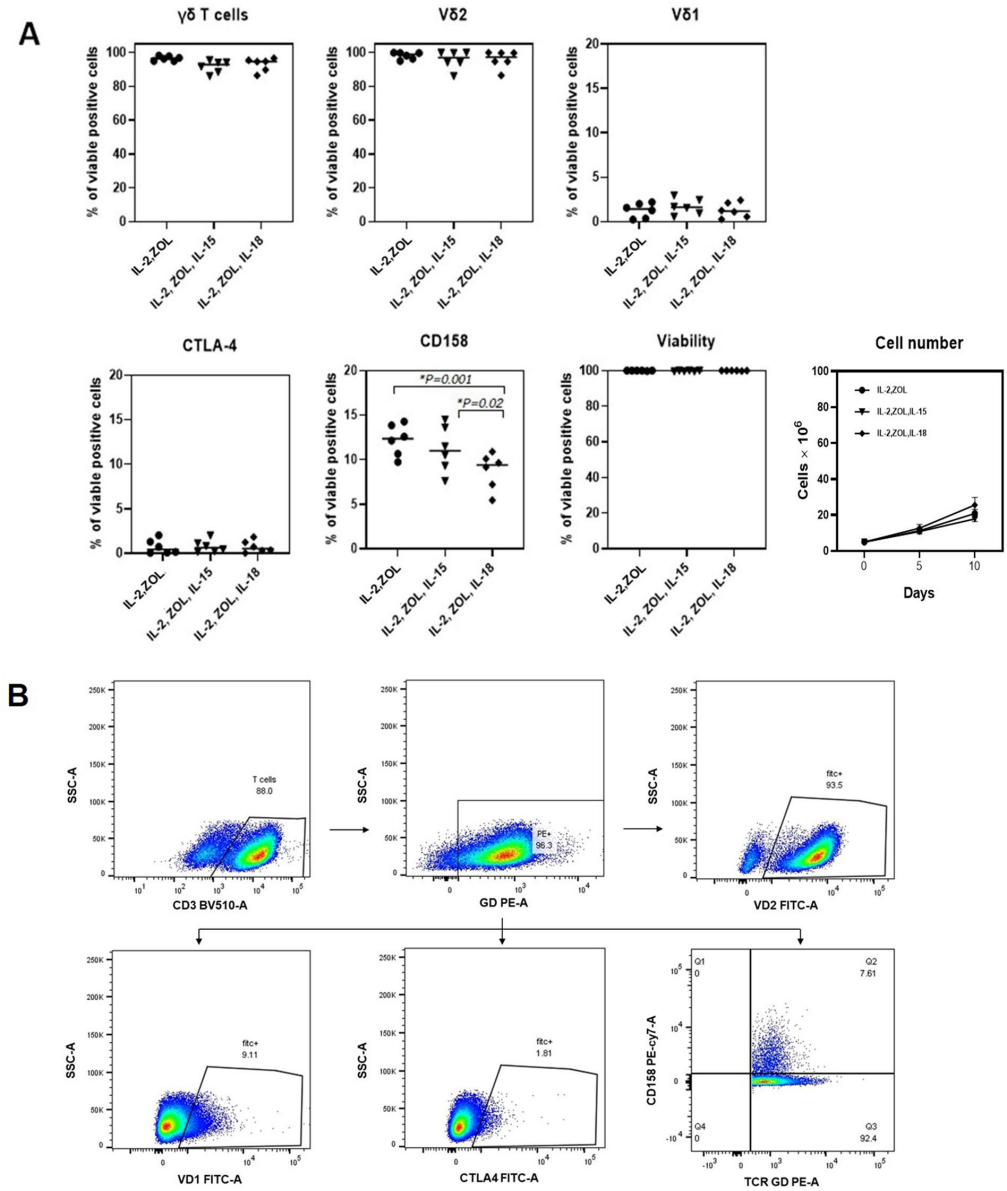
Immunophenotyping and viability of ex-vivo expanded Vγ9Vδ2 T cells. PBMCs from 6 healthy donors were cultured for 10 days with zoledronate and IL-2, and IL-15 or IL-18 or neither. The cells after culture were stained and analyzed for T cell markers by flow cytometry. (**A**) Data shown are the percentage of cells expressing T cell receptor markers including γδ, Vδ2 and Vδ1, co-inhibitory markers CTLA-4 and CD158 with viability determined by 7AAD staining and cells number. The lines represent the mean values. (**B**) Representative flow cytometry panels with the percentage of the Vγ9Vδ2 T cells after cultivation with zoledronate, IL-2, and IL-15.

PBMC from healthy donors were cultured simultaneously with one of three different conditions: complete medium containing 5μM zoledronate and 200 IU IL-2/ml alone, or with 30 ng IL-15/ml, or with 30 ng IL-18/ml or with neither. All subsequent flow cytometry measurements were performed after gating on γδ T cells. Assessments with flow cytometry at the end of cultivation for phenotype and viability indicated that all three protocols resulted in high numbers of viable Vδ2^+^ T cells (IL-2 = 96.53% ± 0.55, IL-15 = 91.68% ± 1.56, IL-18 = 93.00% ± 1.61). The number of cells in these three groups which increased with time in culture can reflect the efficiency of the protocols in proliferating the cells. Following the purification method, isolated cells consisted of > 98% Vγ9Vδ2 T cells as determined by FACS analysis (representative result out of 6 different experiments in each three protocols). Interestingly, zoledronate and IL-2, without either IL-15 or IL-18, provided the best results with regards to a high percentage of Vδ2^+^ T cells with significantly high percentage of CD158, also known as KIRs (killer cell immunoglobulin-like receptors) (IL-2 = 12.07% ± 0.78/IL-2,Zol,IL-15 = 10.34% ± 0.38/IL-2,Zol,IL-18 = 7.93 ± 0.69) [MD, 95%CI between IL-2 and IL-2,Zol,IL-18 = 4.14, 2.22–6.06, *p* = 0.001, and between IL-2,Zol,IL15 and IL-2,Zol,IL-18 = 2.41%, 0.48–4.33, *p* = 0.02] (Fig. 1A). Moreover, no significant differences were observed in the expression of the CTLA4 on γδ T cells stimulated with the different protocols.

### Analysis of cytokine gene expression

**Figure 2:**
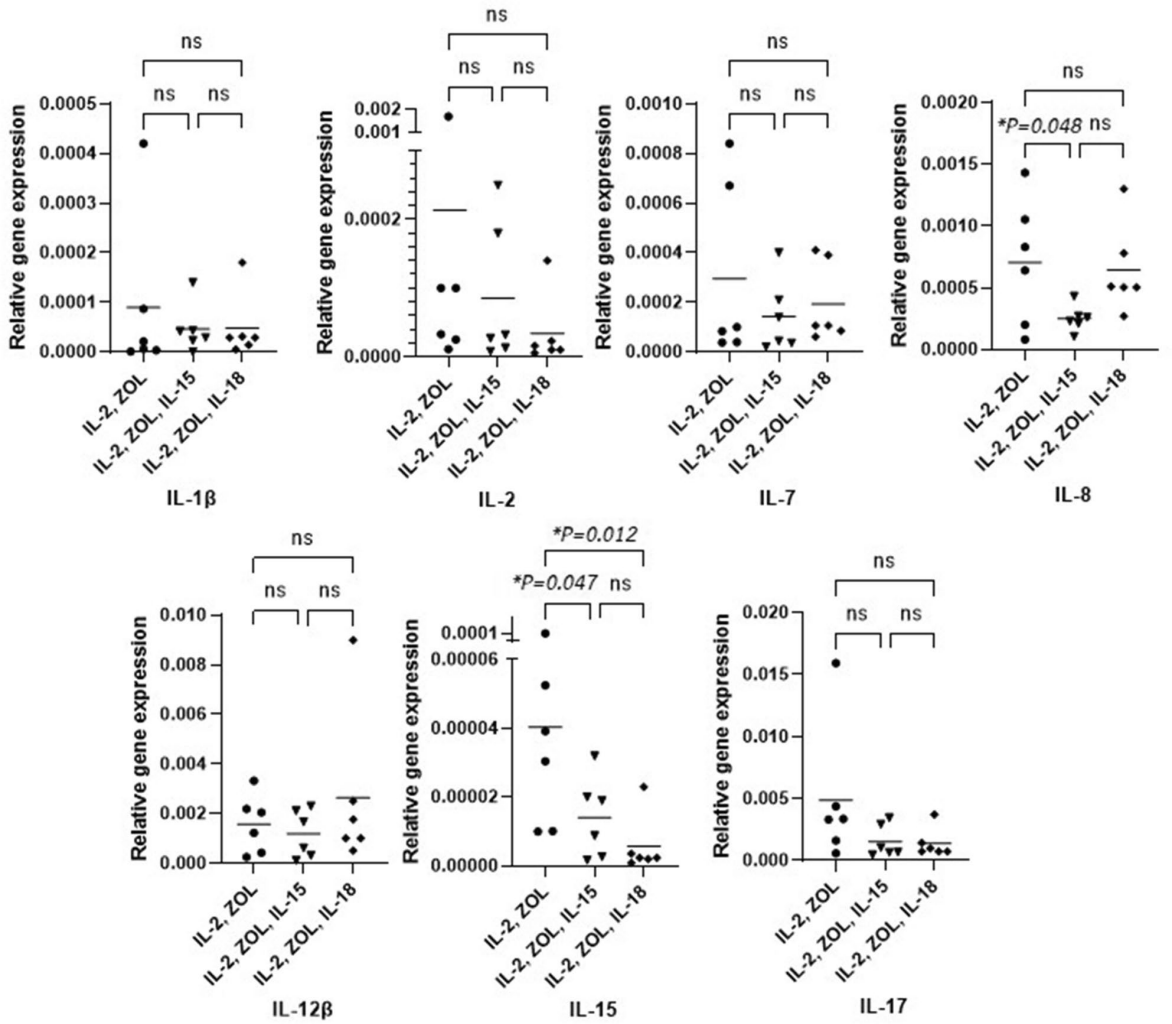
Real-time PCR analysis of cytokine gene expression in expanded Vγ9Vδ2 T cells. Vγ9Vδ2 T cells from 6 healthy donors were propagated as described and analyzed for mRNA expression levels coding for IL-1β, IL-2, IL-6, IL-7, IL-8, IL-12β, IL-15 and IL-17. Data displayed are the results for all tested cases. The lines represent the mean values.

Vγ9Vδ2 T cells were subsequently analyzed for their expression of the cytokine genes IL-1β, IL-2, IL-6, IL-12β, IL-15, and IL-17 by using real-time PCR. Expression of all the cytokine genes expression could be observed with all three cultivation protocols. The determination of the gene expression pattern of Vγ9Vδ2 T cells cultivated from peripheral blood of all healthy donor indicated there were only IL-8 gene expression with significantly increased in condition with IL-2 alone in compared with IL-2,Zol,IL-15 (IL-2 = 0.0007 ± 0.0005/IL-2,Zol,IL-15 = 0.00025 ± 0.0001) [MD, 95%CI = 0.0005, 0.00004 ± 0.0009, *p* = 0.048] as well as IL15 gene expression with significantly increased in condition with IL-2 alone in compared with IL-2,Zol,IL-15 and IL-2,Zol,IL-18 (IL-2 = 0.00004 ± 0.0003/IL-2,Zol,IL-15 = 0.00001 ± 0.00001/IL-2,Zol,IL-18 = 0.00001 ± 0.00001) [MD, 95%CI between IL-2 and IL-2,Zol,IL-15 = 0.00003, 0.0001–0.00005, *p* = 0.047] [MD, 95%CI between IL-2 and IL-2,Zol,IL-18 = 0.00003, 0.0001–0.00006, *p* = 0.012]. However, there were no significantly differences among our three protocols in IL-1b, IL-2, IL-6, IL-7, IL-12b and IL-17. This might be implied that cultivation of Vγ9Vδ2 T cells supplemented with those supportive cytokines led to the same quality of Vγ9Vδ2 T cells (Fig. 2).

### Analysis of apoptotic profile of Vγ9Vδ2 T cell cultures

**Figure 3:**
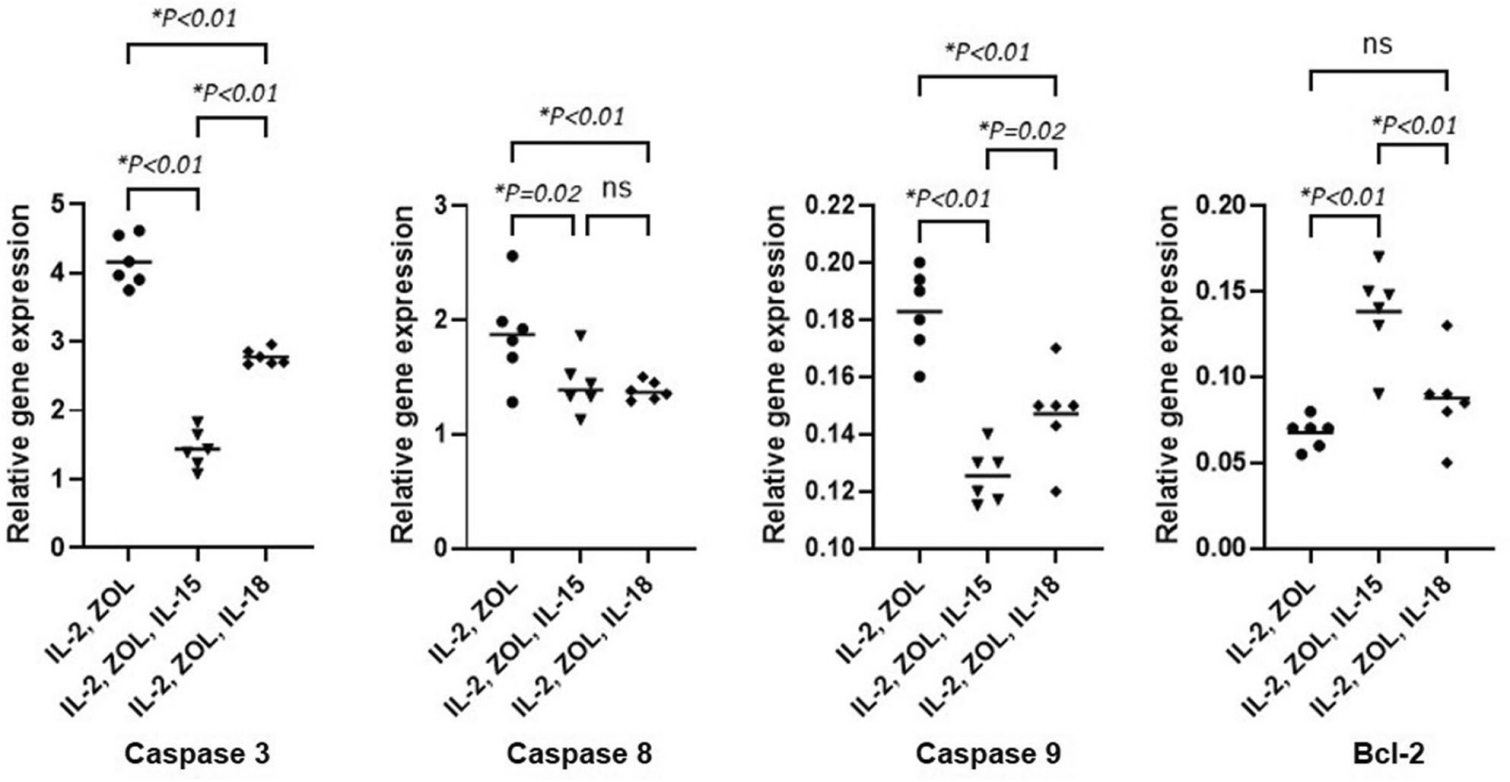
Real-time PCR analysis of the pro- and anti-apoptotic gene expression in expanded Vγ9Vδ2 T cells. Vγ9Vδ2 T cells from 6 healthy donors were propagated as described and analyzed for mRNA expression levels coding for caspase 3, caspase 8, caspase 9 and bcl-2. Data displayed are the results for all tested cases. The lines represent the mean values.

To investigate whether different protocols for generation of Vγ9Vδ2 T cells result in differential pro- and anti-apoptotic signaling, the mRNA levels of various signaling molecules were analyzed. From the real-time PCR results, expression of pro-apoptotic signals, especially caspase 3, was significantly decreased in the IL-15 and IL-18 stimulation protocol when compared to IL-2 protocol (Mean ± SD of IL-2 = 4.16 ± 1.45/IL-2,Zol,IL-15 = 1.44 ± 0.11/IL-2,Zol,IL-18 = 2.78 ± 0.46) [MD, 95%CI between IL-2 and IL-2,Zol,IL-15 = 2.72, 2.32–3.12, *p* < 0.01, and between IL-2 and IL-2,Zol,IL-18 = 1.38%, 0.89–1.79, *p* < 0.01]. Caspase 8 was significantly decreased in the IL-15 and IL-18 (Mean ± SD of IL-2 = 1.87 ± 0.17 / IL-2,Zol,IL-15 = 1.43 ± 0.10 /IL-2,Zol,IL-18 = 1.38 ± 0.33) [MD, 95%CI between IL-2 and IL-2,Zol,IL-15 = 0.44, 0.11–0.87, *p* = 0.02, and between IL-2 and IL-2,Zol,IL-18 = 0.49%, 0.41–1.84, *p* < 0.01]. Caspase 9 was significantly decreased in the IL-15 and IL-18 (Mean ± SD of IL-2 = 0.18 ± 0.01/IL-2,Zol,IL-15 = 0.13 ± 0.01/IL-2,Zol,IL-18 = 0.15 ± 0.01) [MD, 95%CI between IL-2 and IL-2,Zol,IL-15 = 0.06, 0.41–0.74, *p* < 0.01, and between IL-2 and IL-2,Zol,IL-18 = 0.36%, 0.19–0.52, *p* < 0.01]. BCL-2 was significantly increased in the IL-15 but not significantly difference in IL-18 (Mean ± SD of IL-2 = 0.07 ± 0.01/IL-2,Zol,IL-15 = 0.14 ± 0.01 /IL-2,Zol,IL-18 = 0.09 ± 0.01) [MD, 95%CI between IL-2 and IL-2,Zol,IL-15 = 0.07, 0.04–0.98, *p* < 0.01] (Fig. 3).

### Cholangiocarcinoma cells treated with zoledronate enhanced cytotoxicity of Vγ9Vδ2 T cells

**Figure 4:**
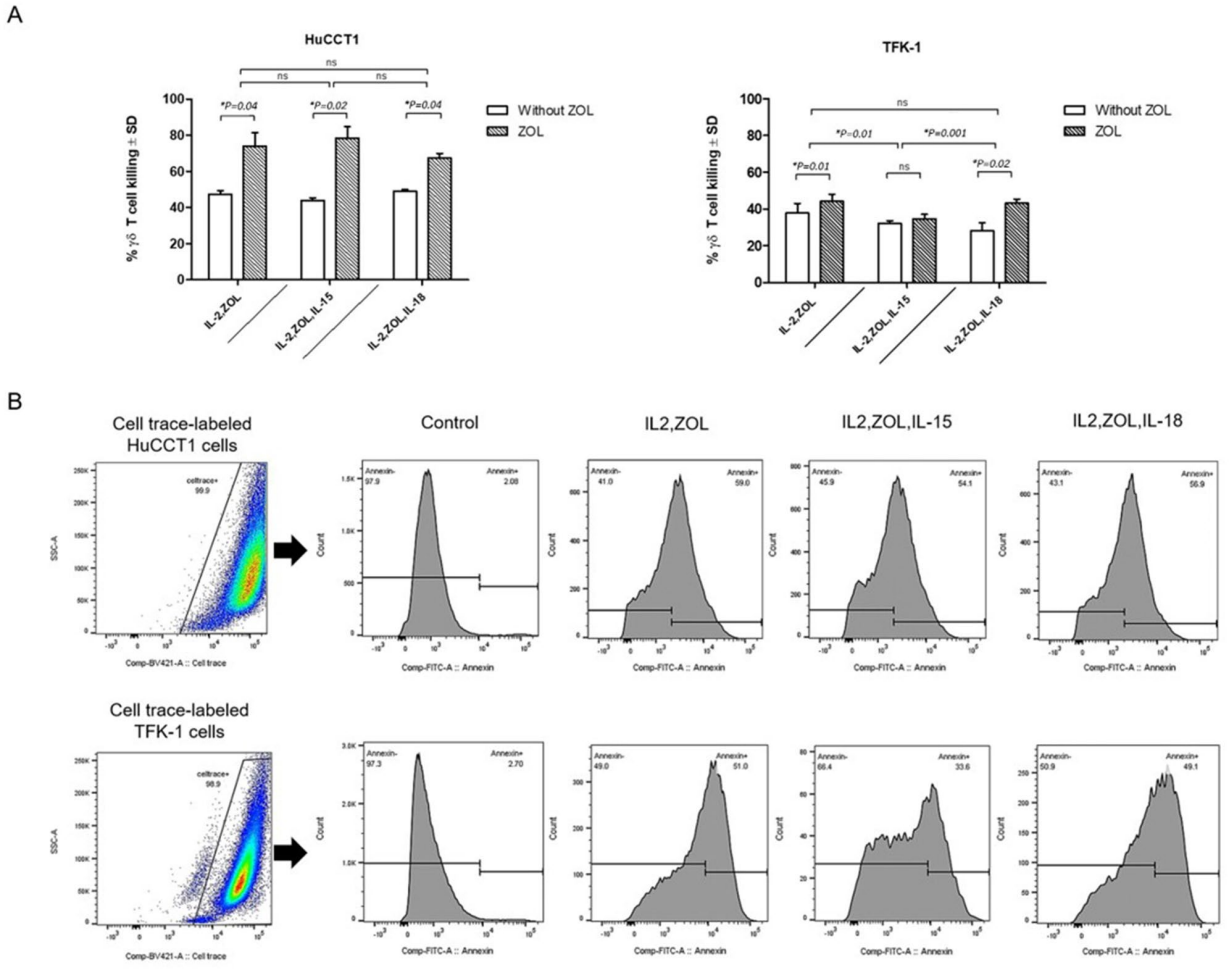
Cytotoxicity of Vγ9Vδ2 T cells against cholangiocarcinoma cells. Cytotoxic properties of cultivated Vγ9Vδ2 T cells against HuCCT1 and TFK-1 cholangiocarcinoma cell lines are displayed. (**A**) Percentages of apoptotic cells at 10:1 E:T ratio are shown, comparing addition or non-addition of zoledronate for 24 h. Data shown are the mean values of cytotoxicity ± SD from the 3 independent experiments performed with different Vγ9Vδ2 T cell lines. (**B**) Representative flow cytometry panels with gating for Cell trace-labeled tumor cells and histogram plots of Annexin V^+^ gated cells from a control (cholangiocarcinoma cell lines without Vγ9Vδ2 T cells) and co-cultures of cholangiocarcinoma cell lines and Vγ9Vδ2 T cells.

The cytotoxic activity of expanded Vγ9Vδ2 T cells from healthy donors against cholangiocarcinoma cell lines was tested. Both of the cell lines, HuCCT1 and TFK-1, were efficiently killed by Vγ9Vδ2 T cells. HuCCT1 cells were more susceptible to kill by Vγ9Vδ2 T cells compared to TFK-1 cells. Treatment with zoledronate for 24 h was sufficient to render both HuCCT1 and TFK-1 cell lines highly susceptible to Vγ9Vδ2 T cell killing (E:T ratio of 10:1). Data showed increasing levels of cytotoxicity in zoledronate treated HuCCT1 compared with non-treated cells in three protocols (Mean ± SD of IL-2 = 74.02 ± 12.80, *p* = 0.04/ IL-2,Zol,IL-15 = 78.36 ± 11.20, *p* = 0.02 /IL-2,Zol,IL-18 = 67.35 ± 4.47, *p* = 0.04) (Fig. 4A). Moreover, the cytotoxic activity towards both zoledronate treated HuCCT1 and TFK-1 target cells of Vγ9Vδ2 T cells were compared. The Vγ9Vδ2 T cells from IL-15 stimulation protocol apparently kill TFK-1 target cells significantly less than the other groups (Mean ± SD of IL-2 = 44.14 ± 3.99/IL-2,Zol,IL-15 = 34.59 ± 2.50/IL-2,Zol,IL-18 = 43.15 ± 2.25) [*P-value* between IL-2 and IL-2,Zol,IL-15 = 0.01, and between IL-2,Zol,IL-15 and IL-2,Zol,IL-18 = 0.001]. In the same experiments, the E:T ratios were varied as 1:1, 5:1, 10:1, 20:1 to demonstrate the cytotoxic activity towards cholangiocarcinoma cells of Vγ9Vδ2 T cells. At E:T ratios of 1:1 and 5:1, the cytotoxic activity of Vγ9Vδ2 T cells did not shift significantly whereas at E:T ratios of 20:1, similar results as 10:1 were obtained with expanded Vγ9Vδ2 T cells derived from healthy donors.

### Killing of cholangiocarcinoma cells mediated by CD107 pathway

**Figure 5:**
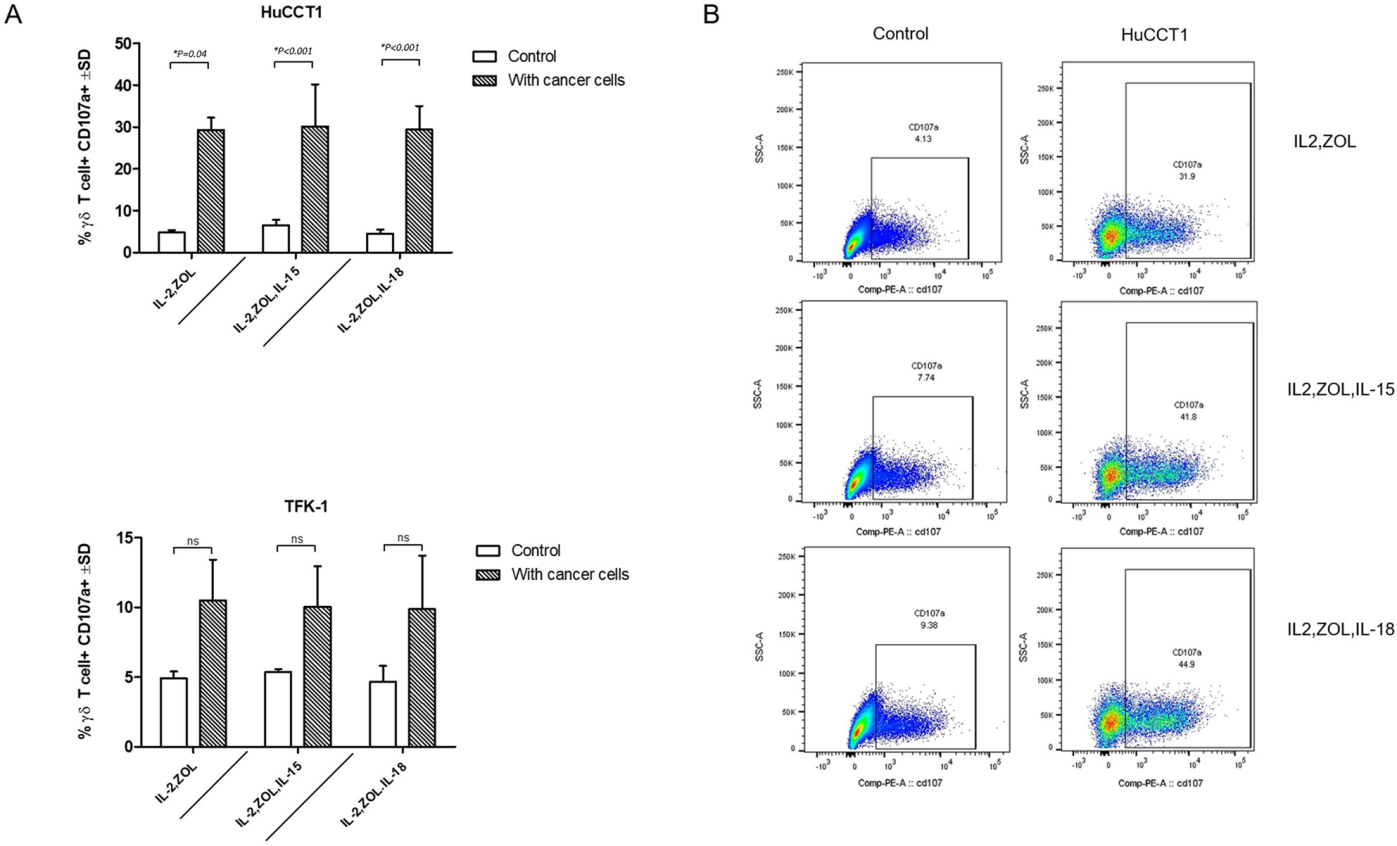
Percentage of Vγ9Vδ2 T cells expressing surface CD107a. (**A**) Percentage of CD107^+^ Vγ9Vδ2^+^ T cells at a given E:T ratio are shown as compared with control. Data shown are the mean values the mean ± SD from 3 independent experiments performed with different Vγ9Vδ2 T cell lines. (**B**) Representative flow cytometry panels with gating for CD107^+^ Vγ9Vδ2^+^ T cells, between inclusion and non-inclusion of a cholangiocarcinoma cell line after 4 h.

To determine possible mechanisms involved in Vγ9Vδ2 T cell-mediated cytotoxicity, we examined cell surface expression of a lysosomal-associated membrane protein (LAMP-1). Also known as CD107a, this is an integral membrane protein localized within cytolytic granules transiently mobilized to the surface of the cell during degranulation. As shown in Fig. 5A, the average expression of CD107a on the surface of Vγ9Vδ2 T cells exposed to both HuCCT1 and TFK-1 cell lines were higher than control, especially in HuCCT1 in our three protocols (Mean ± SD of IL-2,Zol = 29.29 ± 2.96, *p* = 0.04/IL-2,Zol,IL-15 = 30.20 ± 10.05, *p* < 0.001/IL-2,Zol,IL-18 = 29.40 ± 5.60, *p* < 0.001). Based on these initial findings, both cholangiocarcinoma cell lines can activate the degranulation process of Vγ9Vδ2 T cells.

## Discussion

Human Vγ9Vδ2 T cells, a major subset of γδ T cells in peripheral blood, have received increased interest regarding this immune response to cancer over the past decade. Clinical trials utilizing Vγ9Vδ2 T cell-based cancer immunotherapy have been launched and their efficiency to lyse a broad range of tumor cells has been reviewed^27–29^. Given that T cell receptors (TCRs) of Vγ9Vδ2 T cells can recognize antigens in a non-MHC restriction manner, these cells can be generated as adoptive allogeneic cells therapy with low chance to cause graft-versus-host disease (GVHD)^30,31^. Indeed, with the APC property, Vγ9Vδ2 T cells can bridge between innate and adaptive immune systems and lead to other immune cell types providing anti-tumor function^32^. Altogether, Vγ9Vδ2 T cell-based cancer immunotherapy provide the advantages over other forms of cancer treatment. It is known that Vγ9Vδ2 T cells acquaint isopentenyl diphosphate (IPP, also called phosphoantigens), intermediates of the mevalonate pathway, via their TCRs^33,34^. In order to obtain large numbers of Vγ9Vδ2 T cells from PBMCs, nitrogen-containing bisphosphonate drugs (N-BPs, synthetic drugs commonly used for the treatment of postmenopausal osteoporosis) can be used to indirectly activate Vγ9Vδ2 T cells. This occurs through inhibition of farnesyl diphosphate (FPP) synthase and yields in IPP accumulation in human monocytes^35–37^. Zoledronate, a third-generation N-BP, has been shown to stimulate Vγ9Vδ2 T cells relying upon farnesylated proteins. The in vitro proliferation of Vγ9Vδ2 T cells activated by zoledronate combined with IL-2 allows a promising strategy for both in vitro and clinical studies. The feasibility and clinical safety of Vγ9Vδ2 T cells therapy have been evaluated^31,38^. Unknown is whether Vγ9Vδ2 T cells have immunosuppressive function or proinflammatory cytokines releasing which may exert some side effects or risks of using these cells in cancer treatment. The effective way to manage would be identifying and depletion of these cells from the adoptive cell product before infusion in clinical trial^31,39^.

Normally, IL-2 exerts T lymphocytes proliferation and differentiation into effector T cells and memory T cells^40–42^. Vγ9Vδ2 T cell expansion can be furthered by the addition of various cytokines, such as IL-15, which act via Toll-like receptors (TLRs) as reviewed by Wesch, et al*.*^43^. IL-15 is an important cytokine involved in induction of T cell proliferation and cytotoxic activity^44^. It was reported that the greater proliferative and cytotoxic capacities of γδ T cells were resulted from the addition of IL-15 into γδ T cell cultures^45^. IL-18 is also specifically known for its role in inducing cell-mediated immunity. The functional effects of IL-18 on human intraepithelial lymphocyte (IEL) proliferative responses have been reported^46,47^. However, the stimulatory effects of IL-18 on γδ T cells remains unclear. In this study, three different stimulation protocols for Vγ9Vδ2 T cell cultures were compared: one protocol using only recombinant IL-2, one using IL-2 with additive IL-15, and one using IL-2 with additive IL-18. In all of the cultivation protocols, zoledronic acid was used as a stimulant of phosphoantigens. Cultivation with all three protocols resulted in γδ T cells predominantly expressing Vδ2 TCRs as shown in Fig. 1A, in concordance with previous studies^45,48^. Interestingly, there is significant difference in co-inhibitory markers CD158 expression among three groups, suggesting that supportive cytokines can augment the proliferative responses of Vγ9Vδ2 T cells.

Normally, variety protocols for cultivation of Vγ9Vδ2 T cells have been launched mostly based on cytokine-based stimulation method. Different protocols might result in different Vγ9Vδ2 T cells persistent and this might be the importance issue to concern since the existence of cultivated Vγ9Vδ2 T cells will affect the yield and purity of the cultivation for further analysis experiment significantly. Therefore, we identified the molecular mechanisms of cytokine-based stimulation protocols. Regarding cytokine production by Vγ9Vδ2 T cells, highly elevated mRNA levels of cytokines needed for T cell proliferation and differentiation were detected in all protocols. These types of cytokines were reported to specify a preferential development of a cytokine-producing phenotype of Vγ9Vδ2 T cells. However, there is no significant difference among three protocols which might suggest that cultivation of Vγ9Vδ2 T cells supplemented with supportive cytokines, IL-15 and IL-18 led to the same phenotype of Vγ9Vδ2 T cells. In addition, the significant differences of cytokine gene expression were found in umbilical cord blood and peripheral blood γδ T cells from previous studies suggesting the capable of generating of cytokine-producing phenotype in umbilical cord blood γδ T cells^49,50^. We also analyzed for pro-apoptotic gene expression in every cultivation protocol. Interestingly, there were with significantly lower expression of caspase 3, caspase 8 and caspase 9 in both IL-15 and IL-18 cultivation protocols (Fig. 3). This knowledge regarding cytokine-based stimulation protocols may increase the therapeutic efficacy of Vγ9Vδ2 T cells, especially where the additional stimulation with IL-15 and IL-18 might be useful in generating very high numbers with high persistency of Vγ9Vδ2 T cells. Our data is in concordance with previous studies^45,46^.

To investigate the in vitro cytotoxicity of Vγ9Vδ2 cells against cholangiocarcinoma, ex vivo-expanded Vγ9Vδ2 T cells from all cultivation methods were assessed. Our study clearly demonstrated that purified and ex vivo-expanded Vγ9Vδ2 T cells can kill cholangiocarcinoma cell lines and pretreatment of target cells with zoledronate further activated the cytotoxicity of Vγ9Vδ2 T cells in vitro (Fig. 4A) which is similar to previous reports^51,52^. According to the accumulation of IPP within zoledronate-treated cancer cells, expanded Vγ9Vδ2 T cells expressed higher cytotoxic functions against zoledronate-treated cholangiocarcinoma cell lines compared to non-treated cells. Taken together, cultivation based on IL-15 or IL-18 stimulation should be considered when appropriate persistency and anti-tumor functions of Vγ9Vδ2 T cells are required. Exploration of the protocol here used, combining zoledronate and IL-2 with addition of IL-15 or IL-18, led to a substantial induction and proliferation of Vγ9Vδ2 T cells.

The hallmark of Vγ9Vδ2 T lymphocytes aimed to kill tumors is their histocompatibility unrestricted cytotoxic ability and their potentiality to secrete cytokines involved in the anti-tumor response^38,53,54^. Direct cell contact-dependent lysis is other important antitumor effect mediated by Vγ9Vδ2 T cells^52,55,56^. As a general note, the specific cytotoxic potential of cytotoxic T lymphocytes can be described by the detection of CD107a expression, a LAMP-1 dwelling in cytolytic granule membranes located within the cytoplasm. CD107a transiently expressed and mobilized to the effector T cells surface following stimulation thus providing cytotoxic functional readout^57–59^. The mechanisms of cytotoxic activity of Vγ9Vδ2 T cells against cholangiocarcinoma cells are important to better understand. We observed high levels of CD107a expression on Vγ9Vδ2 T cell surfaces after exposure to tumor cell lines. This suggested that potential Vγ9Vδ2 T cell targeting of cholangiocarcinoma might be via a perforin-dependent cytolytic pathway. Our observation is in line with the findings of a previous study^60^. In addition, other possible mechanisms involved in the induction of cholangiocarcinoma apoptosis by Vγ9Vδ2 T cells need to be studied. According to the ongoing identification of new targets for immunotherapy in cholangiocarcinoma, a T cell basis has been developed for improving outcomes in patients with cholangiocarcinoma. Of note, with the antigen cytolytic capacity, allogenic γδ T cells have demonstrated safety and antitumor efficacy in cholangiocarcinoma patient with no adverse effect from treatment.^21,30,61,62^. Hence, Vγ9Vδ2 T cells could be a good candidate for development as an effective treatment of patients with this type of lethal disease.

## Conclusions

Taken together, data from our study support the addition of the immunostimulatory cytokines IL-15 and IL-18 to in vitro γδ T cell cultures as a feasible and efficacious approach to activation and expansion of Vγ9Vδ2 T cells. This may offer the good approach to achieve higher yields of expanded γδ T cells with suitable characteristics for consideration for use in clinical trials. In seeking more specific effector cells for adoptive therapeutics against cholangiocarcinoma, we demonstrated that Vγ9Vδ2 T cells can mediate cytotoxicity against cholangiocarcinoma and that zoledronate can be used to sensitize cholangiocarcinoma cells to this cytotoxic activity. In this regard, Vγ9Vδ2 T cells may help facilitate development of novel strategies for adoptive immunotherapy in cholangiocarcinoma.

## Materials and methods

### Cholangiocarcinoma cell line

Two human cholangiocarcinoma cell lines, HuCCT1 and TFK-1, were used HuCCT1 (TKG0389) and TFK-1 (TKG0367) were purchased from Ricken Cell Bank. Both cell lines were grown in RPMI 1640 medium (Hyclone, USA) supplemented with 10% FBS (Department of Transfusion Medicine, Karolinska University Hospital, Huddinge, Sweden), 100 IU/mL penicillin G and 100 mg/mL streptomycin (Gibco, USA). Cells were incubated at 37 °C in 5% CO_2_ and passaged in T75 flasks once the cells reached 90% confluence.

### Ex vivo expansion of Vγ9Vδ2 T cells

PBMCs, obtained from six peripheral blood buffy coats of healthy donors, were separated by density gradient centrifugation using Lymphoprep™ (AXIS-Shied PoC AS, Oslo, Norway). Cells were cultured at a concentration of 1 × 10^6^ viable cells/mL in RPMI 1640 supplemented with 10% pooled human AB serum (Department of Transfusion Medicine, Karolinska University Hospital) and antibiotics in the presence of 5 µM zoledronate (Novartis Pharma) and 500 U/mL recombinant IL-2. After that, cultivated cells were treated with 30 ng/mL IL-15 or 30 ng/mL IL-18. All cell cultures were performed at 37 °C under 5% CO_2_. Viable cells were counted using trypan blue exclusion every other day and re-plated to maintain cell concentration. At day 10 of cultivation, γδ T cells were separated using a γδ T cell separation kit (Miltenyi Biotech, Bergisch Gladbach, Germany) according to the manufacturer’s instructions and checked subsequently for purity using flow cytometry. The study was approved by the ethical committees of the Karolinska University Hospital in Huddinge, Stockholm, Sweden. Informed consent was obtained from all patients according to the Karolinska Institute and with the declaration of Helsinki.

### Immunophenotyping by flow cytometry

After cultivation of Vγ9Vδ2 T cells, cell surface staining was performed. Briefly, cells were incubated at 4 °C for 15 min with a combination of antibodies as follows before flow cytometric (FACS) analysis: anti-CD3-BV510 (UCHT1), anti-CD8-APC-cy7 (SK1), anti-CD27-BV421 (M-T271) (BD Biosciences, USA), anti-CD4-Alexa fluor 700 (RPA-T4), anti-TCR Vγ9-FITC (B3), anti-TCR Vδ2-FITC (B6), anti-CD45RA-PE (HI100), anti-PD-1-PE (EH12.2H7), anti-CD158-PE-cy7 (HP-MA4) (BioLegend, USA) and anti-CD152/CTLA4-FITC (A3.4H2.H12) (LifeSpan Biosciences, USA). Acquisition was performed on a FACSCANTO flow cytometry instrument using the FACSDiva software (BD Biosciences, USA). The acquired data was analyzed with FlowJo software (Tree Star, Inc., Ashland, OR). Stained control samples were used for gating according to the fluorescence minus one technique.

### Real-time polymerase chain reaction (PCR) analysis

Real-time PCR of cytokine and apoptotic gene expressions (mRNA) were performed with the expanded γδ T cells which had been cultured with 5 μM zoledronate and 500 IU IL-2/mL, and stimulated with 30 ng/mL IL-15 or 30 ng/mL IL-18 cytokine or neither. After cultivation, cells were collected and RNA extracted using the PureLink™ RNA Mini Kit (Thermo Fisher Scientific, USA). cDNA was generated using the SuperScript™ IV VILO™ Master Mix (Thermo Fisher Scientific, USA) according to the manufacturer’s instructions. RNA and cDNA were checked for purity using the NanoDrop Spectrophotometer (Thermo Fisher Scientific, USA). Real-time PCR was performed on an ABI 7500 fast real-time PCR instrument (Applied Biosystems, USA). Relative gene expression of IL-1B, IL-2, IL-6, IL-7, IL-8, IL-12B, IL-15, and IL-17 was measured using pre-formulated TaqMan Gene Expression Assays and the TaqMan BACTIN Gene Expression Control Kit (Applied Biosystems, USA) as a previously published method^49^. Relative gene expression of caspase 3, caspase 8, caspase 9 and bcl-2 was measured using the PowerUp™ SYBR™ Green Master Mix Kit (Applied Biosystems, USA). All measurements were performed in duplicate.

### Cytotoxic assay

To test the killing activity of the cultured Vγ9Vδ2 T cells, a cytotoxicity assay was performed as previously described^49^. Purified Vγ9Vδ2 T cells were re-suspended at a final concentration of 5 × 10^6^ cells/mL; 200 µL was then added to round-bottom polystyrene tubes together with cholangiocarcinoma cells (100 µL) to obtain an E:T ratio of 10:1. Target tumor cells, both HuCCT1 and TFK-1, were labeled with the CellTrace™ Violet Cell Proliferation Marker (CTV, Thermo Fisher Scientific, USA) prior to co-cultivation. Briefly, a total of 10 mL of 2 µM CellTrace™ reagent was added to target tumor cells. Cells were incubated for 20 min at 37 °C. After incubation, cells were washed twice with culture medium containing FBS. Cells were then trypsinized to pellet target cells. Control tubes containing only labeled target cells were also prepared to establish background levels of cell death. Tubes, with or without 5 µM zoledronate, were gently mixed and centrifuged at 1700 rpm for 2 min, and incubated at 37 °C in 5% CO_2_ for 24 h. At the end of the incubation period, 3 µL Annexin V was added to each tube and placed in the dark for 15 min. Finally, 200 µL PBS was added before acquisition using a FACSCANTO flow cytometry. The calculation of cytolytic activity was calculated utilizing the following equation:

$$\% \, \upgamma \updelta {\text{ T cell killing}} = {1}00 - \left( {\% {\text{ viable CTV}} + {\text{tumor cells in co-cultures with }}\upgamma \updelta {\text{ cells}}} \right)/\left( {\% {\text{ viable CTV}} + {\text{tumor cells in control culture without }}\upgamma \updelta {\text{ cells}}} \right) \times {1}00.$$

### CD107 assay

A CD107 assay, with the Vγ9Vδ2 T cells and cholangiocarcinoma cell lines, was set up according to the manufacturer’s instructions (BD Biosciences, USA) and a previously published method^63^. The cholangiocarcinoma cells, HuCCT1 and TFK-1 cell lines, were plated at 1 × 10^6^ cells/mL in round-bottom polystyrene tubes and the Vγ9Vδ2 T cells were added to the tubes for E:T ratios of 10:1. Control tubes containing only Vγ9Vδ2 T cells were also set up and run with each assay. One microliter of GolgiStop (BD GolgiStop™ Protein Transport Inhibitor) and 20 µL of anti-CD107a-PE (H4A3) were added to each tube. Tubes were then incubated at 37 °C under 5% CO_2_ for 4 h. At the end of the incubation period the cells were harvested and stained with anti-CD3-BV510 (UCHT1) and anti-TCR Vδ2-FITC (B6) for 15 min. Cells were then washed again and re-suspended in PBS and analyzed using flow cytometry.

### Statistical analyses

All data were analyzed using the SPSS program (PASW Statistics program 18.0). The mean difference was compared by using compared mean and One-Way ANOVA with Least Significant Difference (LSD) test. The results were considered statistically significant (*p* < 0.05) at the 95% confidence interval. The data shown as Mean ± Standard Error. The mean difference between values shown as mean difference (MD), 95% confidence interval, *P-value*.

## Data Availability

All data generated or analysed during this study are included in this published article.
